# Comparison of outcomes between 20, 23 and 25 gauge vitrectomy for idiopathic macular hole

**DOI:** 10.1186/s40942-015-0007-6

**Published:** 2015-06-26

**Authors:** Fatma Dihowm, Mathew MacCumber

**Affiliations:** 1 Graduate College, Rush University Medical Center, Chicago, IL USA; 2 Department of Ophthalmology, Rush University Medical Center, Chicago, IL USA

**Keywords:** Idiopathic macular hole, Internal limiting membrane (ILM) peeling, 20, 23, 25 gauge vitrectomy, Perfluoropropane (C3F8), Sulfur hexafluoride (SF6)

## Abstract

**Purpose:**

To compare the results of 20, 23, 25 gauge pars plana vitrectomy (PPV) with two different gas tamponades for idiopathic macular hole (MH) in a multi-surgeon vitroretinal practice.

**Methods:**

In this comparative, retrospective, interventional case series, the medical charts of 142 eyes/130 patients were reviewed. Patients who matched our inclusion criteria: eye with stage 2, 3, or 4 MH that underwent 20, 23, or 25 gauge PPV, internal limiting membrane (ILM) peeling, and fluid-gas exchange from January, 2005 to May, 2012 and had at least 6 months follow-up. The best current corrected visual acuity (VA) and anatomical status of the MH were assessed by optical coherent tomography (OCT) at 6 months, 1 year, and 2 years after vitrectomy.

**Results:**

The MH closed successfully after primary vitrectomy in 86.5 % (20 gauge), 96.4 % (23 gauge), and 92 % (25 gauge). Preoperative VA median were 20\126 (20 gauge), 20\100 (23 gauge), and 20\80 (25 gauge). At 6 months and 2 years postoperative VA did not differ significantly between the 3 groups (p = 0.570, and 0.054 respectively). However, at 12 months postoperative VA median 20\60 (20 gauge), 20\69 (23 gauge), and 20\40 (25 gauge) differ significantly (p = 0.005) likely due to cataract changes. The final median postoperative VA (at 2 years) in 25 gauge PPV group was 20/40 which was better than final visual outcomes for 20, and 23 gauge PPV groups (20/50, and 20/55 respectively). The different was not a statistically significant. MH closed successfully in 96 % (C3F8), and 88.1 % (SF6) (p = 0.063). Preoperative median VA was 20/100 in both groups of gas. At 6 months, 1 year, and 2 years postoperative median VAs did not differ significant between the 2 groups (p = 0.076, 0.343, and 0.309 respectively). MH closed successfully in (96.9 %) 12-14 % C3F8, and (95.3 %) 15-16 % C3F8 (p = 0.611). MH closed in (82.1 %) 18-20 % SF6, and (96.4 %) 22-26 % SF6 (p=0.053).

**Conclusion:**

Based on the results of this study, 20, 23, and 25 gauge of PPV have similar MH closure rates and VA outcomes. SF6 at 22-26 % or C3F8 at 12-14 % achieved maximum closure rates.

## Background

Idiopathic macular hole (MH) is relatively common cause of visual loss, with women having about double the rate of men. Although the pathophysiology of MH is not fully known, it is strongly associated with certain factors such as the presence of anteroposterior and tangential traction [1]. Idiopathic full-thickness macular hole has an incidence of approximately 1 per 1000 population causing variable reduction of vision [2]. The natural course of MH leads to a loss of central vision, which stabilizes between 20/200 and 20/400 [1]. Pars plana vitrectomy (PPV) with intravitreal gas tamponade has been the established treatment for MH since it was first described by Kelly and Wendel in 1991. Surgery comprises a three-port PPV combined with induction of a posterior hyaloid detachment if not already present, removal of the posterior cortical vitreous, and intraocular tamponade with long-acting gas followed by a period of face-down positioning. PPV relieves the antero-posterior and tangential traction responsible for induction and maintenance of the foveal dehiscence [3, 4]. Repair of MH was originally achieved by performing 20-gauge PPV with removal of adherent cortical vitreous, stripping of epiretinal membranes followed by gas-fluid exchange, and prone positioning for at least 1 week [5]. Modifications in PPV instruments have led to a decrease in gauge and consequently smaller incisions [6]. A 25-gauge vitrectomy system with sutureless self-sealing sclerotomies was first introduced and found it to be safe surgical procedure in a variety of viteoretinal pathologies [6, 7]. In 2005, Eckardt promoted the 23-gauge transconjunctival system, targeted at combining the benefits of 25- and 20-gauge instrumentation, as a way to overcome some of disadvantages of 25-gauge system at the time [8]. Because of its well-known intraoperative and postoperative advantage, that is, reduction of surgical time and postoperative inflammation, and less pain, small-gauge transconjunctival vitrectomy is gaining wide acceptance [7]. Modifications have been proposed to increase the rate of closure, such as the use of:- different gases (sulfur hexafluoride [SF_6_]; hexafluoroethane [C2F6]; octoflouropropane [C3F8]) and variation in the post-operative time for facedown positioning. Other advances have been internal limiting membrane (ILM) peeling with or without dye to further relieve traction on the hole [9], and the use of adjuncts such as autologous serum and fibrin, transforming growth factor beta, platelets, and thrombin to aid in the wound healing process [2]. Despite of all these developments, there has been little research comparing functional and anatomical efficacy of three gauge PPV systems when used for treating MH. The purpose of this study is to evaluate the anatomical and functional outcomes of these systems for MH management when employ by a multi-surgeon practice, Illinois Retina Associates. All members of this practice are on faculty in Department of Ophthalmology at Rush University Medical Center. The findings can help to optimize surgical outcomes by comparing success rate and complications for 20, 23, and 25 gauge PPV using various concentrations of sulfur hexafluoride (SF6) or perfluoropropane (C3F8).

## Methods

### Study design

This study was a comparative, retrospective, interventional case series. The medical charts of 142 eyes of 131 patients were reviewed. This study was approved by the Rush University Medical Center institutional review board. All eyes that underwent PPV for MH from January, 2005 to May, 2012 and had at least 6 months of follow-up were included. Inclusion criteria included all eyes with stage 2, 3, or 4 MH (Gass classification) [10] and [11] that underwent 20, 23, or 25 gauge PPV, ILM peeling, and fluid-gas exchange. Exclusion criteria included myopia higher than 8D, epiretinal membrane, previous retinal diseases, traumatic MH, and any past history of vitreoretinal surgery. The best current correction of visual acuity (VA) and anatomical status of the MH as assessed by optical coherent tomography (OCT) were examined at the preoperative visit, 6 months, 1 year and 2 years after PPV. All eyes underwent complete preoperative and postoperative slit-lamp biomicro-scopy examination, VA, and dilated fundus examination.

Data collected included age, sex, gender, MH stage, ethnicity, preoperative VA, preoperative lens status, retinal status, postoperative lens status, retinal status of other eye, MH status, VAs at 6 months, 1 year, and 2 years, and any intra or postoperative complications.

### The surgical procedure was as follows

Surgery was performed under local or general anesthesia. All patients underwent PPV with 20, 23, or 25 gauge, standard 3-port approach. View of the posterior segment was achieved with binocular wide-field viewing system or contact lens. A core vitrectomy was performed. Posterior vitreous separation was created (in stage 2 and 3 MH) using the vitreous cutter on aspiration only. The posterior hyaloid was peeled, and the vitreous gel trimmed to the periphery. Indocyanine green (ICG) dye was then infused into eye (in 137 eyes/ 96.5 %) to stain vitreous and ILM. A pick, Tano scraper and/ or microforceps were used to peel the ILM around the MH in all cases. Peripheral retinal examination via microscope or indirect ophthalmoscopy was performed. Indirect or endolaser photocoagulation was applied to any peripheral retinal tears or suspicious retinal lesions (e.g., lattice degeneration, retinal tuft, and peripheral retinal breaks). Fluid-gas exchange was performed in all cases and 12 %-16 % C3F8 or 18 %-26 % SF6 was used as tamponade. Subconjunctival antibiotic and dexamethasone were generally given.

Patients were advised to maintain maximal face down positioning for 1-2 weeks postoperatively. No adjuvants were used during surgeries. Postoperative antibiotic, prednisolone acetate, and atropine eye drops were given to all patients as standard care at our institution.

During postoperative evaluations, patients were examined at 1 day, 1 week, 1 month, 6 months, 1 year, and 2 years. Measurement of VAs was recorded. The anatomical closure was determined after surgery by using indirect slit lamp biomicro-scopy and OCT imaging. Any complications were documented.

Primary outcome variables were postoperative VAs at 6 months, 1 year, and 2 years, which was recorded as a Snellen visual acuity and converted to logarithm of minimal angle of resolution units (LogMAR) for statistical analysis. Secondary outcome measure in this study was anatomical closure rate. Comparing these 2 outcomes between 3 groups of vitrectomy (20 vs 23 vs 25 gauge vitrectomy) and between 2 gas groups (C3F8 group vs SF6 group).

### Statistical analysis

SPSS version 18.0 was used for statistical analysis. Descriptive analysis was performed on all variables and their distribution assessed. A Chi-square test was used to compare categorical variables among the groups and Fisher exact test was used when the sample size was small. A non-parametric analysis was selected to compare VA medians among the groups. The difference between the groups was assessed for statistical significance using Kruskal-Wallis test, Mann-Whitney U test as pair wise difference among the groups, and Wilcoxon test was used to estimate differences before and after treatment. The general linear model/ Analysis of Covariance were used to control any confounders. We hypothesized that there would no statistical significant difference in our primary outcome of VAs or secondary outcome of MH closure at 2 years, between 20, 23, and 25 gauge PPV, and between SF6 and C3F8 groups. A P value ≤ 0.050 was considered significant for all tests.

## Results

### Patients demographics and baseline characteristics

Total of one hundred forty two eyes of 130 patients were studied. The mean follow-up duration ± standard deviation (SD) was 27.8 ± 15.2 months (rang, 6-72 month). Most of the patients were Caucasian 73.9 %, others were African-American 16 %, Hispanic 7 %, and Asiatic 2.1 %. ICG dye used during surgery in 137 eyes (96.5 %), Methyline blue in 1 eye (0.7 %), no dye used in 2 eyes (1.4 %), and Triesence used in 2 eyes (1.4 %).

There were 37 eyes in 20 gauge group, 55 eyes in 23 gauge group, and 50 eyes in 25 gauge group. The 3 groups had similar baseline characteristic in terms of age, gender, stage of MH, preoperative VAs, and preoperative lens status (Table 1). Preoperative VA medians were 0.80 (20/126), range 0.30-2.30 (20/40-count fingers at 1 feet) in 20 gauge group, 0.70 (20/100), range 0.18-1.82 (20/30-count fingers at 3 feet) in 23 gauge group, and 0.60 (20/80), range 0.40-1.70 (20/50- count fingers at 4 feet) in 25 gauge group. A total of 31 patients achieved 2 years of follow-up (83.7 %) in 20 gauge group, 30 patients achieved 2 years of follow-up (54.5 %) in 23 gauge group, and 34 patients achieved 2 years of follow-up (68 %) in 25 gauge group.

**Table 1:** Baseline patients demographics for different gauge groups

Parameter	All eyes, n=142	20 Gauge, n=37	23 Gauge, n=55	25 Gauge, n=50	P value
Age, years					
Mean ±SD	67.8±8.5	69±8.6	67±8.3	66.8±8.5	0.263^a^
Range	46-87	47-87	47-86	46-86	
Gender, n, %					
Female	98 (69 %)	27 (73 %)	38 (69.1 %)	33 (66 %)	0.785^b^
Male	44 (31 %)	10 (27 %)	17 (30.9 %)	17 (34 %)	
Preoperative VA					
Median (logMAR)	0.70	0.80	0.70	0.60	0.237^c^
	0.18-2.30	0.30-2.30	0.18-1.82	0.40-1.70	
Range (logMAR)					
Lens Status, n, %	116 (81.7 %)	27 (73 %)	48 (87.3 %)	41 (82 %)	0.229^b^
Phakic	26 (18.3 %)	10 (27 %)	7 (12.7 %)	9 (18 %)	
Pseudophakic					
Stage of MH, n, %	47 (33.1 %)	9 (24.3 %)	22 (40 %)	16 (32 %)	
Stage 2	54 (38 %)	18 (48.6 %)	19 (34.5 %)	17 (34 %)	0.226^b^
Stage 3	36 (25.4 %)	8 (21.6 %)	11 (20 %)	17 (34 %)	
Stage 4					
ICG	137 (96.5 %)	52 (94.5 %)	52 (94.5 %)	49 (98 %)	0.760^b^

Abbreviation: *n* number, *%* percent

^a^ANOVA

^b^Chi-square test

^c^Kruskal-Wallis test

### Visual and anatomical outcomes

In the 20 gauge group, the medians postoperative VAs were 20/110; range, 20/20 to count finger at 2 feet (p = 0.883) at 6 months, 20/60; range, 20/20 to count finger at 2 feet (p = 0.004) at 1 year, and 20/50; range, 20/20 to count finger at 2 feet (p = 0.001) at 2 years. In the 23 gauge group, the medians postoperative VAs were 20/80; range, 20/25 to count finger at 2 feet (p = 0.026) at 6 months, 20/69; range, 20/25 to count finger at 6 feet (p = 0.018) at 1 year, and 20/55; range, 20/20 to count finger at 5 feet (p = 0.001) at 2 years. In 25 gauge group, the medians postoperative VAs were 20/76; range, 20/20 to count finger at 2 feet (p = 0.192) at 6 months, 20/40; range, 20/20 to count finger at 5 feet (p < 0.001) at 1 year, and 20/40; range, 20/20 to count finger at 5 feet (p < 0.001) at 2 years. In the 3 groups the VA improved significantly at 1, and 2 years after PPV compared to preoperative VA.

One year postoperative VA (median) was significantly better than 6 months postoperative VA (median) in 20 gauge PPV, and 25 gauge PPV group but in 23 gauge the VA improvement was not statistically different (Table 2).

**Table 2:** Comparison in VA improvement postoperative 6 months and 1 year, and 1 year and 2 years in each gauge

Gauge	VA at 6 months	VA at 1 year	P value	VA at 1 year	VA at 2 years	P value
20 gauge	20/110	20/60	0.011	20/60	20/50	0.188
23 gauge	20/80	20/69	0.864	20/69	20/55	0.003
25 gauge	20/76	20/40	0.002	20/ 40	20/40	0.268

Two years postoperative VA (median) was significantly better than 1 year postoperative VA (median) in 23 gauge. However, in 20, and 25 gauge PPV groups the VA improvement was not statistical different (Table 2).

**Fig. 1:**
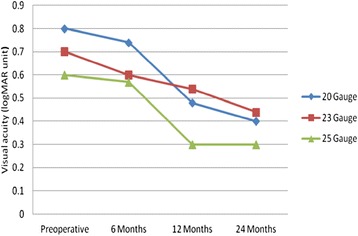
Graph showing preoperative and postoperative logarithm of minimal angle of resolution (logMAR) visual acuity (median) in 20, 23, or 25 gauge vitrectomy systems

Comparison between the 3 groups at 6 months and 2 years, the median VAs did not differ statistically between the 3 groups 20/110, 20/80, and 20/76 at 6 months and 20/50, 20/55, and 20/40 at 2 years, p = 0.570, and p = 0.054 respectively. At 1 year, the median postoperative VAs were statistically different between 20, 23, 25 gauge groups, 20/60, 20/69, 20/40 respectively with p = 0.005. Follow-up tests were conducted to evaluate pairwise differences the 3 groups (comparing 20, 23 gauge group, 20, 25 gauge group, and 23, 25 gauge group), controlling for type I error across the tests among the groups by using Bonferroni approach. The results showed there was no statistically difference between 20 gauge group and 23 gauge group (p = 0.145), and between 20 gauge group and 25 gauge group (p = 0.073). However, the results showed there was statistically significant difference between 23 gauge group and 25 gauge group (p = 0.002) (Fig 1).

**Fig. 2:**
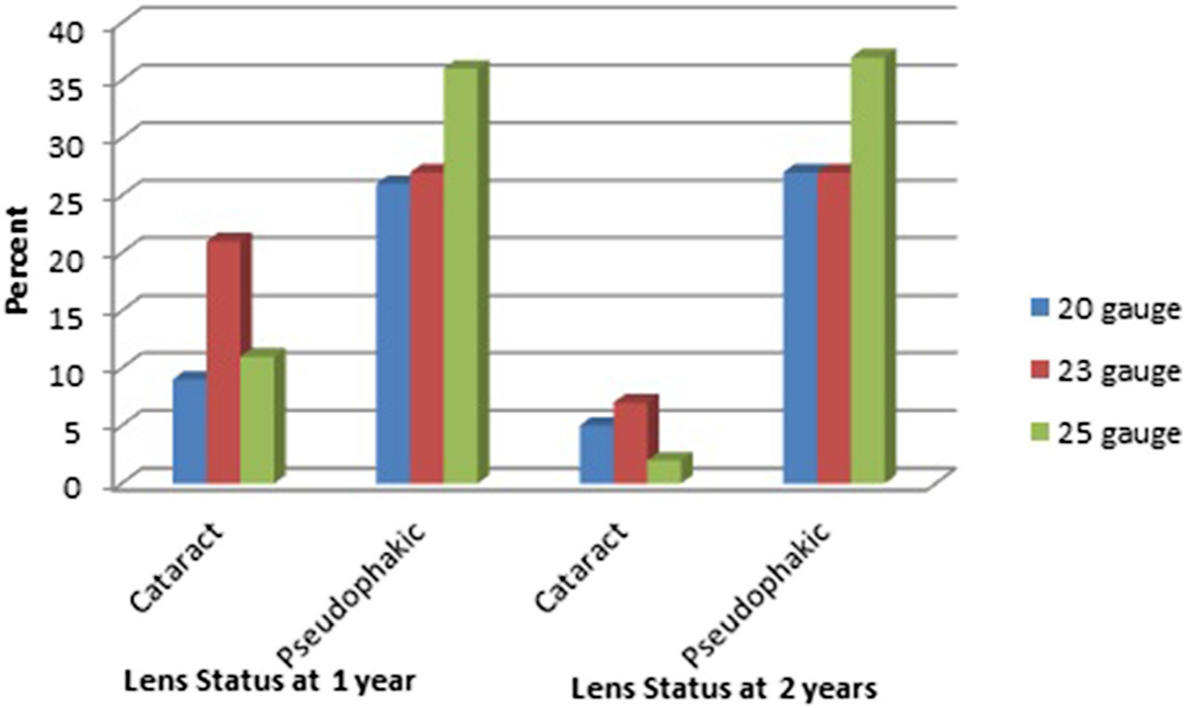
Graph showing Lens Status at 1 and 2 years postoperatively in eyes treated with 20, 23, or 25 gauge PPV systems

General linear model/ Analysis of Covariance conducted for factors that would be expected to bias the visual acuity difference between the 3 groups of vitrectomy at 1 year. The results showed no statistically significant difference (p = 0.136) when the post-operative 1 year lens status was adjusted. Cataract changes were recorded at 1 year after surgery as the following 9 patients (25.7 %) in 20 gauge group, 21 patients (43.8 %) in 23 gauge group, and 11 patients (23.4 %) in 25 gauge group with P = 0.070. At 2 years, 27 patients (84.4 %) were pseudophakic in 20 gauge group, 27 patients (79.4 %) were pseudophakic in 23 gauge group, and 37 patients (94.9 %) in 25 gauge group with P = 0.138 (Fig. 2).

**Fig. 3:**
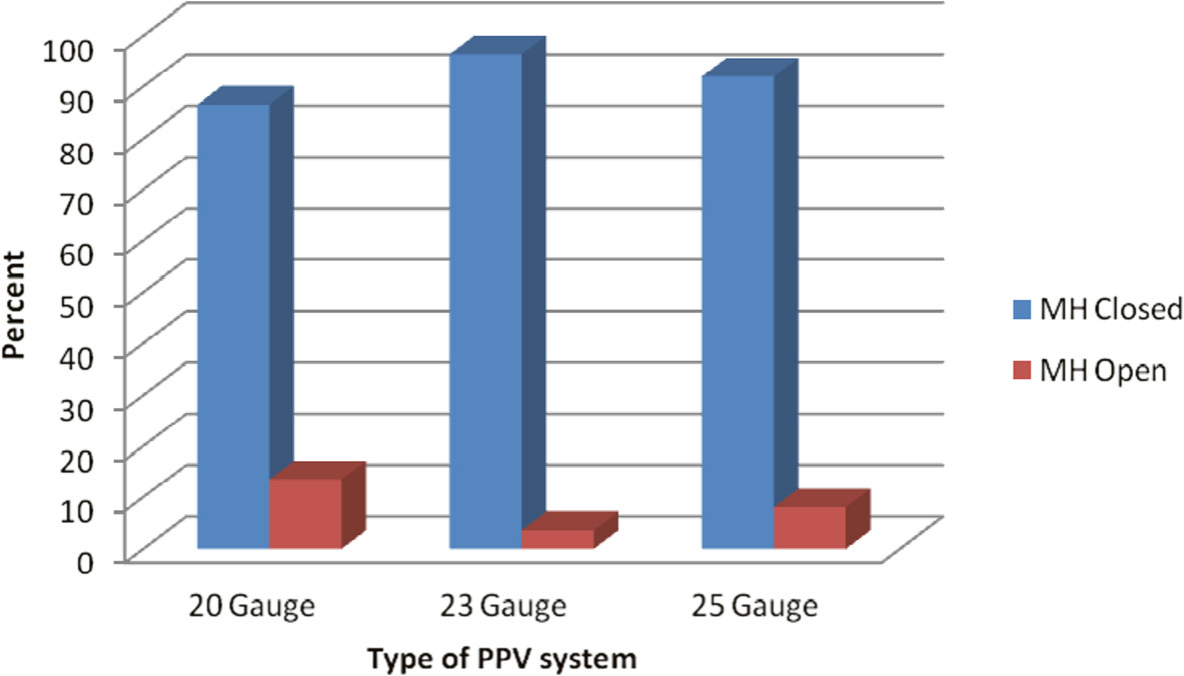
Graph showing Macular hole status in different types of PPV systems after single surgery

Successful primary anatomical closure of MH was achieved in majority of eyes in each of the 3 groups. The MH was closed in 32 of 37 eyes (86.5 %) underwent 20 gauge PPV, 53 of 55 eyes (96.4 %) underwent 23 gauge PPV, and 46 of 50 eyes (92 %) underwent 25 gauge PPV. The difference in MH closure between the 3 groups was not statistically significant (p = 0.220) (Fig. 3).

For the eyes in which MH was closed successfully, VA was 20/63 or better in 63.6 % of 20 gauge PPV group, 55.5 % in 23 gauge PPV group, and 79.4 % in 25 gauge PPV group at 2 years follow-up visit (Table 3). 10.2 % of all eyes which MH closed achieved vision of 20/20, and 71.4 % achieved visual acuity better than 20/64.

**Table 3:** Final VA ranges for different PPV systems in patients who completed 2 years follow-up

VA	20 Gauge	23 Gauge	25 Gauge
20/40 or better	35.7 %	44.1 %	72.2 %
20/41 to 20/63	32.1 %	14.7 %	13.8 %
20/64 to 20/80	10.7 %	14.7 %	5.5 %
Worse than 20/80	21.4 %	26.4 %	8.3 %

### Results of gas groups

Seventy five eyes underwent PPV with injection of 12-16 % C3F8 gas tamponade and 67 eyes underwent PPV with 18-26 % SF6. Baseline demographic variables were compared for any statistical difference including duration of face down positioning in both groups (Table 4).

**Table 4:** Baseline patients demographics for different gas groups

Parameter	C3F8, n= 75	SF6, n= 67	P value
Age, years			
Mean ±SD	69.8±8.3	65.6±8.2	0.003^a^
Range	47-87	46-86	
Gender, n, %			
Female	49/ 65.3 %	49/73.1 %	0.316^c^
Male	26/34.7 %	18/26.9 %	
Preoperative VA			
Median (logMAR)	0.70	0.70	0.381^b^
Range (logMAR)	0.30-2.30	0.18-1.82	
Lens Status, n, %			
Phakic	54/75 %	62/92.5 %	0.002^c^
Pseudophakic	12/28 %	5/7.5 %	
Stage of MH, n, %			
Stage 2	24/32 %	23/34 %	
Stage 3	34/46 %	20/30 %	0.022^c^
Stage 4	12/16 %	24/36 %	
ICG	75/100 %	62/92.5 %	0.055^c^
Duration face-down			
positioning, Mean by Day	9.1	8.5	0.126^a^

Abbreviation: *n* number, *%* percent

^a^T-test

^b^Mann-Whitney U test

^c^Chi-square test and Fisher’s exact test

### Functional and anatomical outcomes for gases

#### Visual acuity in C3F8 versus SF6

The median preoperative VA for eyes treated with C3F8 was 0.70 (20/100), range 0.30 – 2.30 (20/40 to count finger at 1 feet). In eyes treated with SF6 preoperative median was 0.70 (20/100), range 0.18- 1.82 (20/30 to count finger at 3 feet). At 6 months, 1 year, and 2 years, the medians postoperative VA were 20/100, 20/69, and 20/50 in C3F8 group and 20/69, 20/50, and 20/40 in SF6 group. The difference between the 2 groups was statistically not significant (p = 0.076 at 6 months, p = 0.343 at 1 year, and p = 0.309 at 2 years).

### Anatomical outcome of C3F8 versus SF6

**Fig. 4:**
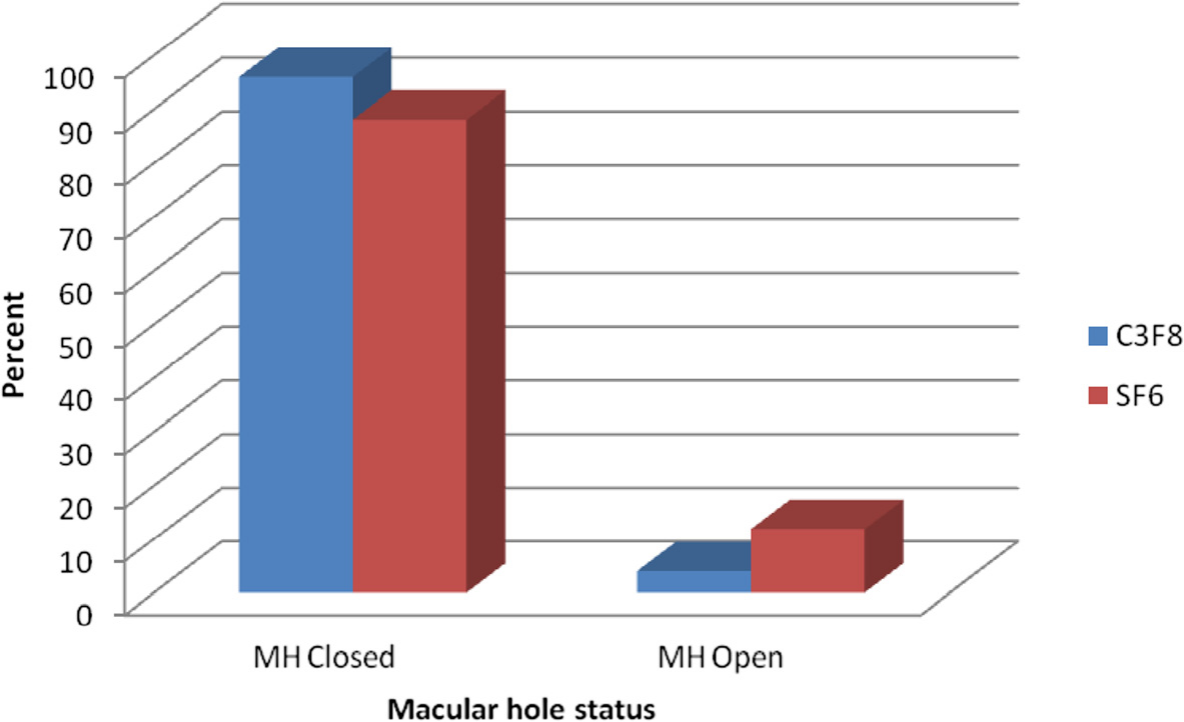
Graph showing macular hole closure rate and type of intraocular tamponade. The overall percentage of eyes with successful MH closure was higher in the eyes treated with Perfluoropropane (C3F8) compared with eyes treated with Sulfur hexafluoride (SF6)

The MH was successfully closed after single surgery with higher rate in long-acting tamponade C3F8 group comparing with short-acting tamponade SF6 group. The MH closed in 72 eyes (96 %) in C3F8 group, and in 59 eyes (88.1 %) in SF6 group, the difference was not statistically significant (p = 0.073) (Fig. 4). Adjusting for age, preoperative VA, stage of MH, duration of face down positioning, and vitrectomy, the difference in success rate between gases remained not significant (P = 0.063).

**Fig. 5:**
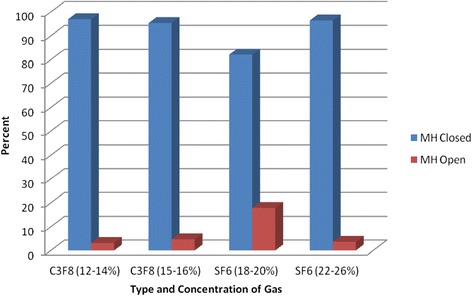
Graph showing macular hole closure and type of intraocular tamponade with different concentrations of gas. The percentage of eyes with successful MH closure was higher in the eyes treated with Perfluoropropane (C3F8) 12-14 %, 15-16 %, and 22-26 % Sulfur hexafluoride (SF6) compared with eyes treated with Sulfur hexafluoride (SF6) at 18-20 %

Subgroup analysis conducted to compare different concentration of each gas. MH closure was achieved in 31 of 32 eyes (96.9 %) treated with 12-14 % C3F8 compared with MH closed in 41 of 43 eyes (95.3 %) treated with 15-16 % C3F8 with (p = 0.611). MH closure rate was compared between eyes which treated with 18-20 % SF6 and eyes treated with 22-26 % SF6. The MH closed in 32 of 39 eyes (82.1 %), and 27 of 28 eyes (96.4 %) respectively with (p = 0.073), although 22-26 % SF6 group had more stage 4 MH than 18-20 % SF6 group it achieved higher closure rate. Logistic regression conducted for the 2 groups of SF6 gas controlling for vitrectomy, MH stage, preoperative VA, and duration of face down postioning. The results showed the gas was only marginally significant (p = 0.053). Comparison between the four groups of gases (12-14 % C3F8 vs 15-16 % C3F8 vs 18-20 % SF6 vs 22-26 % SF6). The difference was significant between the 4 groups with p = 0.048 (Fig. 5).

### Complications

Cataract progression or formation was recorded 67.6 % in 20 gauge group, 70.9 % in 23 gauge group, and 64 % in 25 gauge group. 4 eyes (2.8 %) developed retinal detachment post MH repair. The distribution was in 5.4 % (2 eyes) in the 20 gauge, 1.8 % (1 eye) in the 23 gauge, and 2 % (1 eye) in the 25 gauge. No choroidal detachment, vitreous hemorrhage, or endophthalmitis was recorded in any group.

### Re-operations

Eleven eyes did not close after primary vitrectomy, 8 eyes closed successfully after secondary vitrectomy with fluid-gas exchange, 2 eyes closed after secondary vitrectomy with Silicon oil tamponade, and only one eye suffered closure failure (chronic MH).

## Discussion

Since the original description of MH repair, macular hole surgery has been reported to have increasing success rates, now around 90 % [12]. This study reported MH closure rates of 92.3 % with the ILM peeling, fluid-gas exchange with C3F8 or SF6, and face down positioning matching the success rates published by Rizzo *et al* [13] and Kim *et al* [14]. Primary anatomical closure rate for MH in 23 (96.4 %) and in 25 (92 %) gauge PPV were similar to those reported by Kusuhara *et al* [15]. For 20 gauge PPV group, the MH closure rates (86.5 %) was higher than what Goncu et al [8] reported but lower than what Krishnan *et al* [3] reported. However, comparing MH closure rate between the 3 groups was not statistically significant. This has been reported in other studies but as pair-wise comparison [3, 7, 8, 15]. The results of the present study showed that 20, 23, and 25 gauge PPV would be equally effective techniques for MH management. The final visual acuity increased significantly in the 3 groups in this study. In 20, and 25 gauge PPV group VAs improved at 6 months compared with preoperative but not statistically significantly. At 1 year and 2 years, both groups showed statistically significant improvement in VAs compared with preoperative VAs. On the other hand, in the 23 gauge PPV group VA improved statistically significantly at 6 months, 1 year, and 2 years compared with the preoperative VAs. At one year, postoperative VA (median) was significantly better than 6 months postoperative VA (median) in 20 gauge PPV, and 25 gauge PPV groups. However, in 23 gauge group, the VA improvement was not statistically different. This discrepancy in the results is due to cataract progression or formation.

Analysis of the present study showed that the percentage of patients who underwent cataract extraction in the first year post PPV was higher in the 20 and 25 gauge PPV groups than in the 23 gauge group. The percentage of the patients who were pseudophakic at 1 year was 74.3 % in 20 gauge group, 56.8 % in 23 gauge group, and 76.6 % in 25 gauge group. At 2 years, postoperative VA (median) was significantly better than 1 year postoperative VA (median) in 23 gauge. However, in 20, and 25 gauge PPV groups the VA improvement was not statistical difference compared with postoperative 1 year VAs, most likely because 23 gauge PPV group underwent cataract extraction in the second year post MH repair. Comparing the 3 groups at six months, 1 year, or 2 years postoperatively, final median VAs did not different significantly between the groups (p = 0.570, p = 0.136, and p = 0.054, respectively) matched other study results (pair-wise comparison these studies follow-up period variables from 6 months to 2 years) [3, 7, 8, 15]. The final visual outcomes for the 20, 23, and 25 gauge PPV groups in this study were similar in the 3 groups (20/50, 20/55, and 20/40 respectively) which were better than what Kishnan *et al* [3], and Goncu *et al* [8] have found for 20 and 23 gauge PPV. The better result in current study could be due to better baseline (preoperative) visual acuities compared with the previous two studies, although this study included patients without any limitations for duration of the macular hole in comparison with the previous two studies. The 20 gauge PPV group in this study achieved final median postoperative VA which is consistent with Gupta *et al* results for 132 cases underwent 20 gauge PPV with phacoemulsification and intraocular lens implantation [16]. Moreover, the 23 gauge PPV group in this study had final visual outcome similar to the visual outcome reported by Sanisoglu *et al* [17] in 50 cases of 23 gauge PPV whose a baseline preoperative VA was similar to our group. Furthermore, 25 gauge PPV group achieved better final visual outcome compared with what Carvounis et al reported [5] although the 25 gauge PPV group in our study had similar preoperative VA to Carvounis *et al* study. Finally, in cases of macular holes treated with 25-gauge PPV, Rizzo *et al* documented a median best corrected visual acuity of 20/67 in 77 cases with follow-up range 2 to 24 months [13] which is less than what we report.

Gas tamponade plays an important role in hole closure during macular hole surgery, as the gas provides a scaffold for glial proliferation, and its surface tension may exclude vitreous fluid from the subretinal space [18]. ILM peeling is widely considered to facilitate macular hole closure, perhaps by removing an element of traction or by stimulating gliosis [14]. To maximize the effects of the gas, a long-acting gas and a long duration of face-down positioning have both been used [18], although a majority of vitreoretinal surgeons use C3F8, approximately one-third report using SF6 with excellent results [14, 18].

In this study, the MH closure rate was higher in eyes treated with 12-16 % C3F8 (96 %) than eyes treated with 18-26 % SF6 (88.1 %), however the difference was not statistically significant. Using long-acting gas or short-acting gas as tamponade in MH repair is still controversial, as it has been reported that long-acting gas has better closure rates Thompson *et al* documented [19], while Kim *et al* reported that there was similar MH closure rate between 16 % C3F8 and 20 % SF6 (91 % versus 90 %) [14]. It is difficult to compare the results of our study with results of previously reported studies, because of a difference in gas concentrations for the two gases, and also due to the inclusion of a large number of stage 4 MHs especially in the SF6 group. For example Xirou *et al* [18] used one concentration of each gas, 14 % C3F8 and 20 % SF6, but no stage 4 where included in SF6 group.

Subgroup analysis for C3F8 group showed MH treated with 12-14 % C3F8 achieved closure rate of 96.9 %, and eyes treated with 15-16 % C3F8 achieved closure rate of 95.3 %. No statistical significant difference was found. This is consistent with the results reported by some previous studies [13, 19, 20] and better than [21]. For SF6 group, eyes treated with 18-20 % SF6, MH closed in 83.1 % which was lower compared with eyes treated with 22-26 % SF6 (96.4 %) despite the fact that stage 4 MHs was higher in the second group compared to the first one (39.3 % versus 33.3 %).

Postoperative median VA improved significantly at 6 months in the SF6 group comparing with preoperatively. The C3F8 group did not improve and stayed the same at 20/100. However, the postoperative median VAs at 1 year and 2 years improved in the 2 groups. Our results are similar to previously published studies [14, 18].

Advantages of SF6 include earlier confirmation of closure compared with the C3F8 group and sooner return to air travel and normal daily activities because of more rapid visual recovery commensurate with gas resorption. Closure of macular holes within 3 days after ILM peeling has been reported with confirmation by optical coherence tomography imaging [14]. According to our study, using short-acting 22-26 % SF6 tamponde for MH surgery would be good choice for patients who need rapid visual acuity rehabilitation as it had achieved a similar closure rate to long-acting C3F8 gas tamponade.

Cataract progression or formation is most common complication after MH surgery primarily due to the removal of vitreous and the prolonged exposure to gas [13]. In our study, cataract progression or formation was similar in the 3 groups as most of patients required cataract extraction during the follow-up period. However, in the 25 gauge group, the number of pseudophakic eyes was more than in the other 2 groups. This difference could possibly have be related to the gauge system itself as the 25 gauge system might have allowed more complete removal of vitreous gel around the crystalline lens promoting faster cataract formation compared with the 20 and 23 gauge systems.

There have been several reports about the association between bilateral MH and risk of MH reopening after successful PPV. In our study, 11 bilateral MHs were studied without any reopening of previous closed MH consistent with Passemard *et al* publication [22]. In this study, we recorded 4 cases of post PPV retinal detachement (2.8 %) which was lower than previous studies that reported RD occurrence at rates from 3-14 % [22]. No postoperative cases of endophthalmitis occurred in our study. Limitation of this study are retrospective nature, small sample size especially for the 20 gauge PPV group, missed data (over all we had 23 % of patients data missed at 2 years, in 20 gauge group it was 16.2 %, in 23 gauge group it was 45.4 %, and in 25 gauge group it was 32 %), and a multi-surgeon data set. The varying proficiency of the surgeons could affect the study outcome. However, our results provide useful data that could aid in planning of further prospective, randomized clinical studies.

## Conclusion

In conclusion, based on the results of this study, 20, 23, and 25 gauge of PPV have similar macular hole closure rates and VA outcomes. SF6 at 22-26 % or C3F6 at 12-14 % achieved maximum closure rates.
